# Spike Timing-Dependent Plasticity and Random Inputs Shape Interspike Interval Regularity of Model STN Neurons

**DOI:** 10.3390/biomedicines13071718

**Published:** 2025-07-14

**Authors:** Thoa Thieu, Roderick Melnik

**Affiliations:** 1 School of Mathematical and Statistical Science, College of Health Professions, The University of Texas Rio Grande Valley, Edinburg, TX 78539, USA; thoa.thieu@utrgv.edu; 2 MS2Discovery Interdisciplinary Research Institute, Wilfrid Laurier University, Waterloo, ON N2L 3C5, Canada

**Keywords:** activity-dependent development of nervous systems, spike timing-dependent plasticity, coupled models in medical applications, neuromorphic systems, neurodegenerative diseases, enhanced Hodgkin–Huxley models, Parkinson’s disease

## Abstract

**Background/Objectives:** Neuronal oscillations play a key role in the symptoms of Parkinson’s disease (PD). This study investigates the effects of random synaptic inputs, their correlations, and the interaction with synaptic dynamics and spike timing-dependent plasticity (STDP) on the membrane potential and firing patterns of subthalamic nucleus (STN) neurons, both in healthy and PD-affected states. **Methods:** We used a modified Hodgkin–Huxley model with a Langevin stochastic framework to study how synaptic conductance, random input fluctuations, and STDP affect STN neuron firing and membrane potential, including sensitivity to refractory period and synaptic depression variability. **Results:** Our results show that random inputs significantly affect the firing patterns of STN neurons, both in healthy cells and those with PD under DBS treatment. STDP, along with random refractory periods and fluctuating input currents, increases the irregularity of inter-spike intervals (ISIs) in output neuron spike trains. Sensitivity analyses highlight the key role of synaptic depression and refractory period variability in shaping firing patterns. Combining random inputs with STDP boosts the correlation between neuron activities. Furthermore, at fixed input noise levels, the model’s output closely matches experimental firing rate and ISI variability data from PD patients and animals, with statistical tests confirming significant effects of STDP on firing regularity. **Conclusions:** The findings suggest that the stochastic dynamics of STN neurons, combined with STDP, are crucial for shaping neuronal firing patterns in both healthy and PD-affected states. These insights improve our understanding of how noise and plasticity contribute to neural function and dysfunction, with implications for PD symptom management.

## 1. Introduction

Parkinson’s disease (PD) is a progressive neurodegenerative disorder primarily characterized by the loss of dopaminergic neurons in the substantia nigra pars compacta, which leads to debilitating motor symptoms such as bradykinesia, rigidity, and tremors. Although various treatments, including pharmacological interventions and deep brain stimulation (DBS) of the subthalamic nucleus (STN), have shown efficacy in alleviating these symptoms, the mechanisms underlying these therapies remain poorly understood. Recent studies have highlighted the role of abnormal neuronal oscillations in the STN in contributing to motor dysfunction in PD, and suggested that synaptic plasticity—particularly spike timing-dependent plasticity (STDP)—may play a crucial role in the modulation of these oscillations and the therapeutic effects of DBS [1,2,3].

STDP is a form of synaptic plasticity in which the strength of a synapse is adjusted based on the relative timing of pre- and postsynaptic spikes. This form of learning has been shown to be critical for maintaining neural network stability and promoting coordinated activity [4,5]. In healthy brains, STDP is essential for processes like learning, memory, and sensory processing, but in the context of PD, its dysfunction may contribute to the altered neuronal activity observed in the STN [1,6]. Moreover, the role of STDP in PD pathophysiology remains underexplored, especially with respect to how random factors-such as fluctuations in synaptic inputs or noise from sensory and thermal sources-affect neural firing patterns and network dynamics [7,8]. This gap in understanding motivates our study, which aims to investigate how STDP interacts with stochastic factors in the dynamics of STN neurons. We develop a stochastic model that incorporates both random input currents and synaptic correlation, focusing on their combined effects on the cell membrane potential and neuronal spiking patterns in PD, while several studies have explored the effects of STDP and random inputs in neuronal networks, including modeling studies of the STN [9], as well as studies investigating the mechanistic features of networks with STDP and noise [10,11], the specific interaction between these factors in the context of STN neurons remains underexplored. Additionally, related studies such as those by [12,13] further elucidate the role of noise and plasticity in network dynamics. Burkitt et al. [14] also provide foundational insights into the general mechanisms of STDP in noisy environments, which are relevant for understanding the broader context of our study. Finally, additional work by Lindner [15] offers valuable perspectives on noise-driven network dynamics that complement our investigation. Specifically, we explore how STDP influences the regularity of inter-spike intervals (ISIs) and how random factors such as fluctuations in synaptic inputs or noise from sensory and thermal sources, neural firing patterns and network dynamics [16,17]. This approach is distinct from prior studies, which typically examine STDP in more controlled, deterministic settings [5,18]. Recent studies have explored how STDP and structural plasticity interact under neuromodulatory protocols like coordinated reset (CR) stimulation. These works show that specific stimulation patterns can induce long-lasting desynchronization by leveraging synaptic plasticity. Manos et al. found that short-term, spaced CR stimulation with adaptive parameters can robustly achieve anti-kindling effects even in previously ineffective regimes [19]. Stimulation outcomes vary with frequency and sequence type, with RVS offering robustness and SVS showing stronger effects under optimal settings [20]. Incorporating structural plasticity, CR stimulation was shown to enhance desynchronization after longer stimulation-free periods [21]. Chauhan et al. further demonstrated that combined STDP and structural plasticity demand higher stimulation intensities due to activity-dependent network reorganization [22]. These findings highlight the need to consider both synaptic and structural dynamics in PD models and support our focus on stochastic modulation of STDP in the STN.

Our model builds on well-established frameworks, including the Hodgkin–Huxley (HH) model for single-neuron dynamics [23] and integrate-and-fire (LIF) models for simplifying neuron spiking behavior [24], but uniquely integrates random input fluctuations and STDP to more accurately reflect the stochastic nature of biological neurons. Several studies have explored the interplay between noise and STDP in HH-type neuron models [25] and in LIF models (e.g., [26]). These studies provide important insights into how noise and plasticity together shape neuronal dynamics and learning processes. While previous work has focused on the effects of STDP in healthy networks, our study is one of the first to examine how STDP and random noise interact in the context of PD, a disease characterized by dysregulated neural circuits and irregular neuronal firing [27,28]. Several studies have explored neural networks with STDP and noise in the context of PD. Chauhan et al. (2024) [22] investigated how synaptic weight and structural plasticity co-evolve, revealing how structural reorganization can enhance network synchrony and inform desynchronization strategies for PD. Madadi Asl et al. (2018) [29] showed that incorporating propagation delays and noise in STDP networks allows for bidirectional synapse preservation, offering insights into synaptic dynamics relevant to PD. These studies emphasize the importance of both excitatory and inhibitory inputs, which are crucial for accurately modeling the STN in PD. We build upon the deterministic STN model developed in [30], which was used to study the contributions of local cell and passing fiber activation to thalamic fidelity during DBS and lesioning. While their model considered deterministic dynamics, we introduce a stochastic term to capture the inherent random fluctuations in neuronal activity. These fluctuations, which arise from sensory noise, brainstem discharges, and thermal energy, are crucial for understanding the irregular firing patterns and synaptic plasticity observed in conditions like PD. By extending the model with this stochastic component, we investigate how random inputs influence the neuronal behavior and the efficacy of therapies like DBS.

The main contribution of this work is twofold. First, we propose a novel computational framework that combines STDP with random inputs to model the effects of noise and synaptic plasticity in PD. Second, we explore how these dynamics affect the regularity of neuronal firing, with potential implications for both PD pathophysiology and therapeutic interventions such as DBS. Our results suggest that while STDP can enhance the irregularity of ISIs, the presence of random input currents and refractory periods contributes to the increased variability in spike trains, which may contribute to the motor symptoms observed in PD [5,31]. Our sensitivity analyses highlight the critical roles of synaptic depression and refractory period variability in shaping neuronal output. Additionally, the correlation between the activity of different neurons increases when random inputs and STDP are combined, which further impacts the oscillatory dynamics of the STN. Furthermore, at fixed input noise levels, the model’s output aligns well with experimentally observed firing rates and ISI variability in both PD patients and animals, with statistical analyses confirming that STDP significantly influences spike timing regularity. These findings provide new insights into the neural mechanisms of PD and the potential role of STDP in managing the disease, with implications for future neuromorphic systems designed to mimic these processes [32,33].

## 2. Materials and Methods

STDP is a fundamental mechanism in the brain that modifies the synaptic strengths between neurons based on the coincidence of pre- and postsynaptic spikes. In conventional asymmetric forms of STDP, the temporal order of spikes is critical, so that when the presynaptic spike precedes the postsynaptic spike (i.e., pre–post pairing), the STDP rule leads to long-term potentiation (LTP) of the synapse between pre- and postsynaptic neurons, whereas long-term depression (LTD) is induced in the reverse scenario (i.e., post–pre pairing). Although STDP is a local mechanism and merely depends on the pre- and postsynaptic spike timings, it can determine global connectivity patterns emerging in recurrent neuronal networks.

We will focus on building a model of a synapse in which its synaptic strength changes as a function of the relative timing (i.e., time difference) between the spikes of the presynaptic and postsynaptic neurons, respectively. This change in the synaptic weight is known as STDP. The aims of this paper are to build a model of synapse that show STDP and study how correlations in input spike trains influence the distribution of synaptic weights. We will model the presynaptic input as Poisson-type spike trains. The postsynaptic neuron will be modeled as an HH neuron.

We assume that a single postsynaptic neuron is driven by *N* presynaptic neurons. That is, there are *N* synapses, and we will study how their weights depend on the statistics or the input spike trains and their timing with respect to the spikes of the postsynaptic neuron.

The phenomenology of STDP is typically described by a biphasic exponentially decaying function and order-dependent, with pre–post spiking inducing LTP and LTD [34,35]. The instantaneous change in synaptic weight, ΔW, is given by the following:

(1) $$\begin{array}{cc} \Delta W & =A_{+}e^{\left(t_{\mathrm{pre}}-t_{\mathrm{post}}\right)/\tau_{+}}\;\mathrm{if}\;t_{\mathrm{post}}>t_{\mathrm{pre}}, \end{array}$$

(2) $$\begin{array}{cc} \Delta W & =-A_{-}e^{-\left(t_{\mathrm{pre}}-t_{\mathrm{post}}\right)/\tau_{-}}\;\mathrm{if}\;t_{\mathrm{post}}<t_{\mathrm{pre}}, \end{array}$$

where ΔW represents the change in synaptic weight, and $A_{+}$ and $A_{-}$ determine the maximum extent of synaptic modification. These maximum changes occur when the timing difference between presynaptic and postsynaptic spikes is close to zero. The parameters $\tau_{+}$ and $\tau_{-}$ define the time windows of presynaptic-to-postsynaptic inter-spike intervals that lead to synaptic strengthening or weakening [36]. The latency between presynaptic and postsynaptic spikes (Δt) is defined as follows:

(3) $$\begin{array}{c} \Delta t=t_{\mathrm{pre}}-t_{\mathrm{post}}, \end{array}$$

where $t_{\mathrm{pre}}$ and $t_{\mathrm{post}}$ represent the times of the presynaptic and postsynaptic spikes, respectively. When the postsynaptic neuron fires after the presynaptic neuron (i.e., Δt<0), this leads to a positive change in synaptic weight (ΔW>0).

This model captures the key phenomenon that repeated presynaptic spikes occurring a few milliseconds before postsynaptic action potentials induce long-term potentiation (LTP) of the synapse, whereas presynaptic spikes occurring after postsynaptic spikes result in long-term depression (LTD) at the synapse.

### 2.1. Keeping Track of Pre- and Postsynaptic Spikes

To implement STDP while considering different synapses, we must track the times of both presynaptic and postsynaptic spikes throughout the simulation. To achieve this, we introduce two variables to monitor the spike times: M(t) for the postsynaptic neuron and P(t) for the presynaptic neuron. These variables are used to track the spikes over a longer timescale than the synaptic conductances. For each postsynaptic neuron, we define the following differential equation to track the postsynaptic spike history (see, e.g., [26]):

$$\tau_{-}\frac{dM}{dt}=-M,$$

where $\tau_{-}$ is the timescale over which the postsynaptic spike influence decays. Whenever the postsynaptic neuron spikes, we update M(t) as follows:

(4) $$\begin{array}{c} M\left(t\right)=M\left(t\right)-A_{-}. \end{array}$$

This allows M(t) to track the number of postsynaptic spikes over the timescale $\tau_{-}$, and ensures that M(t) is always negative, which is associated with the induction of LTD. Similarly, for each presynaptic neuron, we define the following differential equation:

$$\tau_{+}\frac{dP}{dt}=-P,$$

where $\tau_{+}$ is the timescale over which the presynaptic spike influence decays. When the presynaptic neuron spikes, we update P(t) as follows:

(5) $$\begin{array}{c} P\left(t\right)=P\left(t\right)+A_{+}. \end{array}$$

Here, P(t) is always positive, corresponding to LTP. The variables M(t) and P(t) play similar roles to synaptic conductances $g_{i}\left(t\right)$, but instead of controlling the synaptic strength, they track the temporal relationship between presynaptic and postsynaptic spikes over much longer timescales. Importantly, M(t) is negative and associated with LTD, while P(t) is positive and associated with LTP, since they are updated by $A_{-}$ and $A_{+}$, respectively.

### 2.2. Implementation of STDP

The peak synaptic conductance of each synapse *i*, denoted by ${\bar{g}}_{i}$, will be adjusted based on the timing of presynaptic and postsynaptic spikes, using the variables M(t) and P(t). The conductance ${\bar{g}}_{i}$ can vary between $\left[0,{\bar{g}}_{\max}\right]$ and is modified according to the relative timing of the spikes [26].

1. When the i-th presynaptic neuron fires a spike, the peak conductance is updated as follows:
  (6) $$\begin{array}{c} {\bar{g}}_{i}={\bar{g}}_{i}+M\left(t\right){\bar{g}}_{\max}.\; \end{array}$$
  Here, M(t) tracks the time since the last postsynaptic spike and is always negative. Therefore, if the postsynaptic neuron spikes shortly before the presynaptic neuron, the peak conductance will decrease, as indicated by the negative value of M(t).
2. When the postsynaptic neuron fires a spike, the peak conductance of each synapse is updated as follows:
  (7) $$\begin{array}{c} {\bar{g}}_{i}={\bar{g}}_{i}+P\left(t\right){\bar{g}}_{\max}. \end{array}$$
  Here, P(t) tracks the time since the last spike of the i-th presynaptic neuron and is always positive.
  Thus, if the presynaptic neuron spikes before the postsynaptic neuron, the peak conductance increases, as indicated by the positive value of P(t).

### 2.3. Leaky Integrate-and-Fire Neuron Connected with Synapses That Show STDP

We connect *N* presynaptic neurons to a single postsynaptic neuron. We do not need to simulate the dynamics of each presynaptic neuron as we are only concerned about their spike times. So, we will generate *N* Poisson-type spikes.

We need to simulate the dynamics of the postsynaptic neuron as we do not know its spike times. We model the postsynaptic neuron as a HH system, which simulates the membrane potential of a STN cell.

Furthermore, motivated by [30,37,38,39], we consider a modified HH system modeling a STN cell membrane potential. In particular, we first choose a STN healthy cell, then switch to a PD cell, and study the effects of random inputs on the STN cell membrane potential under synaptic conductance dynamics.

In biological systems of brain networks, instead of physically joined neurons, a spike in the presynaptic cell causes the release of a chemical, or a neurotransmitter. Neurotransmitters are released from synaptic vesicles into a small space between the neurons called the synaptic cleft [40]. In what follows, we will investigate the chemical synaptic transmission and study how excitation and inhibition affect the patterns in the neurons’ spiking output in our HH model.

In this section, we consider a HH model of synaptic conductance dynamics. In particular, neurons receive a myriad of excitatory and inhibitory synaptic inputs at dendrites. To better understand the mechanisms of synaptic conductance dynamics, we use the description of Poissonian trains to investigate the dynamics of the random excitatory (E) and inhibitory (I) inputs to a neuron [41,42].

We consider the transmitter-activated ion channels as an explicitly time-dependent conductivity $\left(g_{\mathrm{syn}}\left(t\right)\right)$. The conductance transients can be defined by the following equation (see, e.g., [40,41]):

(8) $$\begin{array}{c} \frac{dg_{\mathrm{syn}}\left(t\right)}{dt}=-{\bar{g}}_{i}\sum_{k}\delta \left(t-t_{k}\right)-\frac{g_{\mathrm{syn}}\left(t\right)}{\tau_{\mathrm{syn}}}, \end{array}$$

where ${\bar{g}}_{\mathrm{syn}}$ (synaptic weight) denotes the maximum conductance elicited by each incoming spike, while $\tau_{\mathrm{syn}}$ is the synaptic time constant, and δ(·) is the Dirac delta function. Note that the summation runs over all spikes received by the neuron at time $t_{k}$. We have the following formula for converting conductance changes to the current by using Ohm’s law:

(9) $$\begin{array}{c} I_{\mathrm{syn}}\left(t\right)=g_{\mathrm{syn}}\left(t\right)\left(V\left(t\right)-E_{\mathrm{syn}}\right), \end{array}$$

where *V* is the membrane potential, while $E_{\mathrm{syn}}$ represents the direction of current flow and the excitatory or inhibitory nature of the synapse.

The total synaptic input current $I_{\mathrm{syn}}$ is the combination of both excitatory and inhibitory inputs. Assume that the total excitatory and inhibitory conductances received at time *t* are $g_{E}\left(t\right)$ and $g_{I}\left(t\right)$, and their corresponding reversal potentials are $E_{E}$ and $E_{I}$, respectively. Then, the total synaptic current can be defined by the following equation (see, e.g., [42]):

(10) $$\begin{array}{c} I_{\mathrm{syn}}\left(V\left(t\right),t\right)=-g_{E}\left(t\right)\left(V-E_{E}\right)-g_{I}\left(t\right)\left(V-E_{I}\right)=-I_{E}-I_{I}. \end{array}$$

In [30], the authors have used the quantity $I_{\mathrm{GPe},\mathrm{STN}}$ in the STN model. However, we know that STN-DBS generates both excitatory and inhibitory postsynaptic potentials in STN neurons [43]. In our current consideration, instead of using the current $I_{\mathrm{GPe},\mathrm{STN}}$, we consider the current $I_{\mathrm{STN},\mathrm{DBS}}=-I_{E}-I_{I}$. Let us define the following synaptic dynamics of the STN cell membrane potential (*V*) described by the following model (based on [30])

(11) $$\begin{array}{cc} C_{m}\frac{d}{dt}V\left(t\right) & =-I_{L}-I_{\mathrm{Na}}-I_{K}-I_{T}-I_{\mathrm{Ca}}-I_{\mathrm{ahp}}-I_{\mathrm{STN},\mathrm{DBS}}+I_{\mathrm{app}}+I_{\mathrm{dbs}}\;\;\mathrm{if}\;V\left(t\right)\leq V_{\mathrm{th}}, \end{array}$$

(12) $$\begin{array}{cc} V(t) & =V_{\mathrm{reset}}\;\;\mathrm{otherwise}, \end{array}$$

where $I_{\mathrm{app}}$ is the external input current, while $C_{m}$ is the membrane capacitance and t∈[0,T] (some fixed time *T*). Additionally, in (11), $V_{\mathrm{th}}$ denotes the membrane potential threshold to fire an action potential. In this model, we assume that a spike takes place whenever V(t) crosses $V_{\mathrm{th}}$ in the STN membrane potential. In that case, a spike is recorded and V(t) resets to $V_{\mathrm{reset}}$ value. Hence, the reset condition is summarized by $V\left(t\right)=V_{\mathrm{reset}}$ if $V\left(t\right)\geq V_{\mathrm{th}}$. The quantity $I_{\mathrm{ahp}}$ represents the calcium-activated potassium current for the spike after hyperpolarization in STN.

The concentration of intracellular Ca2+ is governed by the following calcium balance equation

(13) $$\begin{array}{c} \frac{d}{dt}Ca\left(t\right)=\varepsilon \left(I_{\mathrm{Ca}}-I_{T}-k_{\mathrm{Ca}}\mathrm{Ca}\left(t\right)\right), \end{array}$$

where $\varepsilon =3.75\times 10^{-5}$ is a scaling constant, and $k_{\mathrm{Ca}}=22.5$ (${\mathrm{ms}}^{-1}$) is a given time constant (see, e.g., [44,45]).

Furthermore, we consider an external random (additive noise) input current as follows: $I_{\mathrm{app}}=\mu_{\mathrm{app}}+\sigma_{\mathrm{app}}\eta \left(t\right)$, where η is the zero Gaussian white noise with $\mu_{\mathrm{app}}>0$ and $\sigma_{\mathrm{app}}>0$. Using the description of such random input current in our system, the first equation (11) can be considered as the following Langevin stochastic equation (see, e.g., [46]):

(14) $$\begin{array}{cc} C_{m}\frac{d}{dt}V\left(t\right) & =-I_{L}-I_{\mathrm{Na}}-I_{K}-I_{T}-I_{\mathrm{Ca}}-I_{\mathrm{ahp}}-I_{E}-I_{I}\\ & +I_{\mathrm{dbs}}+\mu_{\mathrm{app}}+\sigma_{\mathrm{app}}\eta \left(t\right)\;\;\mathrm{if}\;V\left(t\right)\leq V_{\mathrm{th}}. \end{array}$$

Therefore, the system (11)–(13) (t∈[0,T]) can be rewritten as

(15) $$\begin{array}{cc} C_{m}\frac{d}{dt}V\left(t\right) & =-I_{L}-I_{\mathrm{Na}}-I_{K}-I_{T}-I_{\mathrm{Ca}}-I_{\mathrm{ahp}}-I_{E}-I_{I}\\ & +I_{\mathrm{dbs}}+\mu_{\mathrm{app}}+\sigma_{\mathrm{app}}\eta \left(t\right)\;\;\mathrm{if}\;V\left(t\right)\leq V_{\mathrm{th}}, \end{array}$$

(16) $$\begin{array}{cc} V(t) & =V_{\mathrm{reset}}\;\;\mathrm{otherwise}. \end{array}$$

Furthermore, we consider the following gating variable dynamics (see, e.g., [30])

(17) $$\begin{array}{cc} \frac{d}{dt}h\left(t\right) & =0.75\frac{h_{\infty }\left(V\right)-h\left(t\right)}{\tau_{h}\left(V\right)}, \end{array}$$

(18) $$\begin{array}{cc} \frac{d}{dt}n\left(t\right) & =0.75\frac{n_{\infty }\left(V\right)-n\left(t\right)}{\tau_{n}\left(V\right)}, \end{array}$$

(19) $$\begin{array}{cc} \frac{d}{dt}r\left(t\right) & =0.2\frac{r_{\infty }\left(V\right)-r\left(t\right)}{\tau_{r}\left(V\right)}, \end{array}$$

(20) $$\begin{array}{cc} \frac{d}{dt}c\left(t\right) & =0.08\frac{c_{\infty }\left(V\right)-c\left(t\right)}{\tau_{c}\left(V\right)}, \end{array}$$

(21) $$\begin{array}{cc} \frac{d}{dt}Ca\left(t\right) & =\varepsilon (I_{\mathrm{Ca}}-I_{T}-k_{\mathrm{Ca}}\mathrm{Ca}\left(t\right)). \end{array}$$

The initial data we use for the system (15)–(21) define its initial conditions:

(22) $$\begin{array}{cc} V(0) & =V_{0}, \end{array}$$

(23) $$\begin{array}{cc} h(0) & =h_{\infty }\left(V_{0}\right), \end{array}$$

(24) $$\begin{array}{cc} n(0) & =n_{\infty }\left(V_{0}\right), \end{array}$$

(25) $$\begin{array}{cc} r(0) & =r_{\infty }\left(V_{0}\right), \end{array}$$

(26) $$\begin{array}{cc} c(0) & =c_{\infty }\left(V_{0}\right), \end{array}$$

(27) $$\begin{array}{cc} Ca(0) & =\frac{a_{\infty }\left(V_{0}\right)}{a_{\infty }\left(V_{0}\right)+b_{\infty }\left(V_{0}\right)}, \end{array}$$

where $h_{\infty },n_{\infty },r_{\infty },c_{\infty },a_{\infty },b_{\infty }$ are described as in Table 1.

In our model (15)–(27), as we mentioned above, we use the simplest input spike train with the Poisson process in which the stochastic process of interest provides a suitable approximation to stochastic neuronal firings [47]. The input spikes will be carried out by the quantity $\sum_{k}\delta \left(t-t_{k}\right)$ in Equation (8) and each input spike arrives independently of the others, meaning that the timing of one spike does not influence the timing of subsequent spikes. The process will be described as follows:

- For designing a spike generator of spike train, we define the probability of firing a spike within a short interval (see, e.g., [41]) as $P\left(1\;\mathrm{spike}\;\mathrm{during}\;\Delta t\right)=r_{j}\Delta t$, where j=e,i with $r_{e},r_{i}$ representing the instantaneous excitatory and inhibitory firing rates, respectively.
- Then, a Poisson spike train is generated by first subdividing the time interval into a group of short sub-intervals through small time steps Δt. In our model, we use Δt=0.1 (ms).
- We define a random variable $x_{\mathrm{rand}}$ with uniform distribution over the range between 0 and 1 at each time step.
- Finally, we compare the random variable $x_{\mathrm{rand}}$ with the probability of firing a spike, which reads as follows:
  (28) $$\begin{array}{c} \left\{\begin{array}{c} r_{j}\Delta t>x_{\mathrm{rand}},\;\mathrm{generates}\;a\;\mathrm{spike},\\ r_{j}\Delta t\leq x_{\mathrm{rand}},\;\mathrm{no}\;\mathrm{spike}\;\mathrm{is}\;\mathrm{generated}. \end{array}\right. \end{array}$$

By using model (15)–(27), we also investigate the effects of random refractory periods. We consider the random refractory periods $t_{\mathrm{ref}}$ as $t_{\mathrm{ref}}=\mu_{\mathrm{ref}}+\sigma_{\mathrm{ref}}\tilde{\eta }\left(t\right)$, where $\tilde{\eta }\left(t\right)∼N\left(0,1\right)$ is the standard normal distribution, $\mu_{\mathrm{ref}}>0$ and $\sigma_{\mathrm{ref}}>0$.

In this study, we model the external input to neurons using Poisson spike trains, which are commonly employed in computational neuroscience to simulate random background activity [48,49]. Although real neurons show inter-spike intervals that deviate from the Poisson assumption [50,51], Poisson processes remain a useful tool for isolating the effects of stochastic noise on STDP [52]. Poisson spike trains are simple and effective in providing random, uncorrelated inputs, which allows us to explore the basic dynamics of STDP and noise interactions in the context of PD without introducing additional complexities like input correlations or network-level dynamics.

In general, the information on stimulating activities in a neuron can be provided by the irregularity of spike trains. The time interval between adjacent spikes is called the ISI. The coefficient of variation (CV) of the ISI in a cell membrane potential with multiple inputs can bring useful information about the output of a decoded neuron. In what follows, we will demonstrate that when we increase the value of $\sigma_{\mathrm{ref}}$, the irregularity of the spike trains increases (see also [53]). The term spike irregularity refers to the coefficient of variation of inters-pike intervals, which quantifies the variability in the timing of spikes rather than the variability of the spike count. The spike irregularity of spike trains can be described via the coefficient of variation of the inter-spike-interval (see, e.g., [53,54]) as follows:

(29) $$\begin{array}{c} CV_{\mathrm{ISI}}=\frac{\sigma_{\mathrm{ISI}}}{\mu_{\mathrm{ISI}}}, \end{array}$$

where $\sigma_{\mathrm{ISI}}$ is the standard deviation and $\mu_{\mathrm{ISI}}$ is the mean of the ISI of an individual neuron.

In the next section, let us consider the output firing rate as a function of Gaussian white noise mean or direct current value, namely, the input–output transfer function of the neuron.

In our model, we choose the parameter set as in the following Table 1:

Since these parameters have also been used in [30] for STN cell membrane potential experiments, we take them for our model validation. Moreover, in our consideration, we use not only the parameters from Table 1 but also the following parameters: $V_{\mathrm{th}}=-55$ (mV), $V_{\mathrm{reset}}=-70$ (mV), $V_{0}=-65$ (mV), Δt=0.1, $C_{m}=10$ (nF), $\tau_{E}=2$ (ms), $\tau_{I}=5$ (ms), ${\bar{g}}_{E}=1.5$ (nS), ${\bar{g}}_{I}=0.5$ (nS), $r_{e}=10$, $r_{i}=10$, $n_{E}=20$ spike trains, $n_{I}=80$ spike trains. Here, $n_{E}$ and $n_{I}$ represent the number of excitatory and inhibitory presynaptic spike trains, respectively.

Mathematically, the developed model (15)–(27) is an evolutionary system that combines stochastic differential equations and ordinary differential equations (SDEs-ODEs), where the stochastic membrane potential equation is coupled to the activation and inactivation ion channels equations, as well as to the calcium-activated potassium current equation. This system can be considered as a modified HH system.

### 2.4. Effects of Input Correlations

Correlation or synchrony in neuronal activity refers to the relationship between the firing patterns of different neurons, and it can be measured in various ways. In this context, we focus on the spiking activity of neurons. At its simplest, correlation or synchrony refers to the coincident spiking of neurons—that is, when two neurons spike together, they are said to be firing in synchrony or to be correlated [55]. Neurons can be synchronous in their instantaneous activity, meaning they spike together with some probability. However, synchrony can also occur with a time delay, where the spiking of one neuron at time *t* is correlated with the spiking of another neuron at a later time (time-delayed synchrony). The origins of synchronous neuronal activity consist of the following:

- Common inputs: Neurons receiving input from the same sources tend to have correlated activity. The degree of correlation in their inputs influences the degree of correlation in their outputs.
- Pooling from correlated sources: Neurons may not share the same input neurons but could receive inputs from other neurons that are themselves correlated.
- Direct connections: Neurons connected to each other (either unidirectionally or bidirectionally) can exhibit time-delayed synchrony. Gap-junctions between neurons can also facilitate synchrony.
- Similar properties: Neurons with similar intrinsic parameters and initial conditions may also exhibit synchronous behavior.

When neurons fire together, their coordinated activity can have a stronger influence on downstream neurons. Synapses are sensitive to the temporal correlations between presynaptic and postsynaptic spikes, and this sensitivity plays a crucial role in shaping functional neuronal networks, which is essential for unsupervised learning. While synchrony can reduce the system’s dimensionality, strong correlations may sometimes impair the decoding of neuronal activity. A simple model to study the emergence of correlations involves injecting common inputs into two neurons and measuring the resulting output correlation as a function of the fraction of shared inputs.

In this study, we will investigate how correlations are transferred by calculating the correlation coefficient of spike trains recorded from two unconnected HH neurons that received correlated inputs [55,56]. The input current to HH neurons i=1,2 is given by the following:

$$I_{i}=\mu_{i}+\sigma_{i}\left(\sqrt{1-c}\;\xi_{i}+\sqrt{c}\;\xi_{c}\right),$$

where $\mu_{i}$ represents the temporal average of the current. The Gaussian white noise $\xi_{i}$ is independent for each neuron, while $\xi_{c}$ is common to all neurons. The parameter *c* (where 0≤c≤1) controls the proportion of common and independent inputs, and $\sigma_{i}$ is the variance of the total input. The sample correlation coefficient between the input currents $I_{i}$ and $I_{j}$ is defined as the sample covariance of $I_{i}$ and $I_{j}$ divided by the product of the square roots of their sample variances. Specifically, we use the following equations:

$$r_{ij}=\frac{\mathrm{cov}(I_{i},I_{j})}{\sqrt{\mathrm{var}(I_{i})}\;\sqrt{\mathrm{var}(I_{j})}},$$

$$\mathrm{cov}\left(I_{i},I_{j}\right)=\sum_{k=1}^{L}\left(I_{i}^{k}-{\bar{I}}_{i}\right)\left(I_{j}^{k}-{\bar{I}}_{j}\right),$$

$$\mathrm{var}\left(I_{i}\right)=\sum_{k=1}^{L}{\left(I_{i}^{k}-{\bar{I}}_{i}\right)}^{2},$$

where ${\bar{I}}_{i}$ is the sample mean of $I_{i}$, *k* is the time bin index, and *L* is the total number of samples. Here, $I_{i}^{k}$ represents the current at neuron *i* at time k·dt. Note that these formulas for covariance and variance are not fully accurate as they should be divided by L−1 for sample estimates. However, we omit this correction because it cancels out in the calculation of the sample correlation coefficient.

### 2.5. STDP in Neuromorphic Systems and Other Applications

With many current and potential applications, STDP is often thought of as an unsupervised brain-like learning mechanism for spiking neural networks (SNNs) that, among other things, has attracted significant attention from the neuromorphic hardware community [57,58]. Its ability to mimic biological learning processes makes STDP highly relevant for various applications, including pattern recognition and sensory processing, real-time pattern recognition, stabilized supervised STDP, and synchronization in neural networks. Many models have been proposed to investigate the role of STDP mechanisms across these applications. For example, the human brain is recognized as the most complex entity in the known universe [59]. At the microcircuit level, neuronal cells are organized into layers with various connectivity motifs, while the information processing mechanisms at this level remain not fully understood, investigating these motifs, particularly about STDP, is essential for gaining insights into biological learning mechanisms and the emergence of intelligence. STDP provides plasticity rules that depend on spikes. As such, they are unsupervised learning rules commonly used in spiking SNNs and neuromorphic chips to emulate brain-like information processing. However, a significant performance gap remains between ideal model simulations and their neuromorphic implementations. STDP is implemented in SNNs and neuromorphic chips, serving as an unsupervised learning rule crucial for mimicking brain-like information processing [60]. Its applications include noisy spatiotemporal spike pattern detection, which is particularly effective even with low-resolution synaptic efficacy in neuromorphic implementations. This capability enhances performance in various computational tasks, making STDP relevant for advancing neuromorphic hardware [61,62]. STDP is used to train an efficient Spiking Auto-Encoder that leverages asynchronous sparse spikes for input reconstruction, denoising, and classification, achieving superior performance with significantly fewer spikes compared to state-of-the-art methods. On the other hand, STDP has significant applications in bio-plausible meta-learning models, particularly in enhancing the adaptability and efficiency of machine-learning systems. By incorporating STDP and Reward-Modulated STDP, these models reflect biological learning mechanisms and enable quick learning in low-data scenarios. This approach is particularly useful for preventing catastrophic forgetting in meta-learning tasks, allowing the model to retain previously acquired knowledge while learning new tasks. Additionally, STDP facilitates the application of these models in spike-based neuromorphic devices, improving their performance in tasks such as few-shot classification and advancing AI systems’ capabilities to mimic human-like learning [63]. STDP is employed to train a Spiking Auto-Encoder that efficiently performs input reconstruction, denoising, and classification with minimal spike usage, showcasing enhanced performance on image datasets while maintaining competitiveness against other learning approaches [64]. STDP is also applied through memristors as synapses to enable in situ learning and inference in SNNs. This approach addresses the computational challenges associated with implementing STDP in hardware, allowing for efficient weight modulation that enhances speed and reduces power consumption. The integration of STDP facilitates real-time pattern recognition by employing a winner-takes-all mechanism within the SNN architecture. The proposed design significantly improves performance metrics, including power, energy, and accuracy, enabling the classification of 50 million images per second [65]. STDP is employed in the Stabilized Supervised STDP learning rule to enhance the classification layer of SNNs, integrating unsupervised STDP for feature extraction and improving performance on image recognition tasks [66]. Furthermore, STDP is utilized to investigate the configurations needed to achieve robust synchronization in neural networks, with findings that could inform the design of neuromorphic circuits for improved information processing and transmission through synchronization phenomena [67].

STDP can be regarded as a key learning rule in biological neural networks, and its relevance has been explored in neuromorphic systems for a variety of applications. STDP enables synaptic modifications based on the relative timing of pre- and postsynaptic spikes, which is critical for encoding information and adapting to dynamic environments. Recent advancements in flexible neuromorphic electronics have made it possible to integrate artificial synapses and neurons that replicate the functionality of their biological counterparts, providing a platform for computing, soft robotics, and neuroprosthetics. These systems, which emulate synaptic behaviors and exhibit learning capabilities, are particularly promising for future applications in health monitoring and the Internet of Things [68]. Furthermore, neuromorphic computing algorithms, which draw inspiration from biological learning rules like STDP, are becoming central to the development of more efficient, adaptive, and scalable computing technologies. The ongoing research in neuromorphic hardware and algorithms is setting the stage for new opportunities in both machine learning and real-world applications such as cognitive computing and neuroprosthetics [69].

Overall, despite the challenges in scaling STDP for deeper networks and larger tasks, its biological relevance and versatility highlight its significance in advancing artificial intelligence and neural computation. STDP holds the potential to enhance the performance of robotic systems by allowing them to learn from their environments in real time, reinforcing its critical role in the development of intelligent systems.

## 3. Results and Discussion

In this section, we take a single STN neuron and study how the neuron behaves under random inputs and when it is bombarded with both excitatory and inhibitory spike trains together with the influence of STDP. The numerical results reported in this section have been obtained using a discrete-time integration based on the Euler method implemented in Python 3.0.

**Figure 1:**
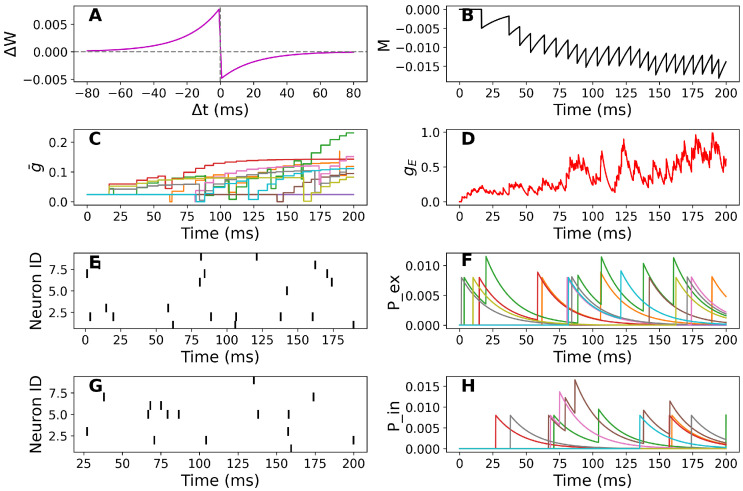
[Color online] (**A**): Example of presynaptic spike trains for excitatory neurons. (**B**): Presynaptic timing (M) dynamics presented in (4). (**C**): Average excitatory synaptic conductance ($\bar{g}$) updates for multiple synapses presented in (6). (**D**): Excitatory synaptic conductance ($g_{E}$) presented in (8). (**E**): Example of presynaptic spike trains for excitatory neurons. (**F**): The effective conductance or strength of synaptic transmission for excitatory neurons ($P_{ex}$) presented in (5). (**G**): Example of presynaptic spike trains for inhibitory neurons. (**H**): The effective conductance or strength of synaptic transmission for inhibitory neurons ($P_{in}$) presented in (5).

In particular, we use the coupled SDEs-ODEs system (8)–(27) that describes the dynamics of the STN membrane potential. In addition, Figure 1A shows the biphasic STDP function used in our model. Synaptic weights are potentiated when the presynaptic spike precedes the postsynaptic spike (Δt<0) and depressed otherwise (Δt>0), with exponential decay governed by time constants $\tau_{\mathrm{STDP}}^{+}$ and $\tau_{\mathrm{STDP}}^{-}$. This rule underlies synaptic updates throughout our simulations. As we have mentioned in the previous section, we will focus on the effects of Gaussian white noise input current together with the random refractory periods on the STN cell membrane potential.

To explore the fundamental behavior of the STN neuron model and gain theoretical insights into its dynamics, we first examine simulation-based results under simplified input conditions. These simulations help us isolate and understand the effects of specific model components such as noise, refractory randomness, and STDP. Subsequently, we adjust the model to accommodate real experimental data and investigate its performance under biologically realistic conditions.

### 3.1. Simulation-Based Results

**Figure 2:**
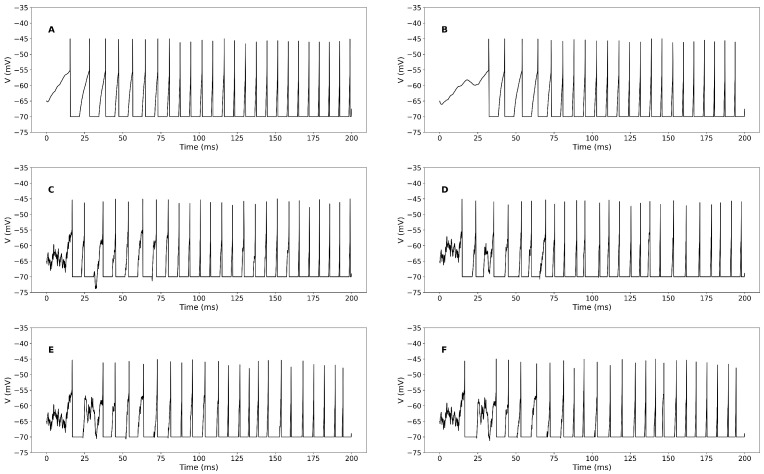
[Color online] Membrane potential profile in STDP case. (**A**): The time-dependent changes in membrane potential and synaptic activity profile of direct input current and direct refractory period of healthy cell. (**B**): The time-dependent changes in membrane potential and synaptic activity profile of direct input current and direct refractory period of PD cell. (**C**): The time-dependent changes in membrane potential and synaptic activity profile of random input current σ=1 and direct refractory period of PD cell. (**D**): The time-dependent changes in membrane potential and synaptic activity profile of random input current σ=1 and random refractory period $\sigma_{\mathrm{ref}}=1$ of healthy cell. (**E**): The time-dependent changes in membrane potential and synaptic activity profile of random input current σ=1 and random refractory period $\sigma_{\mathrm{ref}}=1$ of PD cell. (**F**): The time-dependent changes in membrane potential and synaptic activity profile of random input current σ=1, random refractory period $\sigma_{\mathrm{ref}}=1$ and DBS input current of PD cell. Here, we consider $t_{\mathrm{ref}}=6$ (ms).

**Figure 3:**
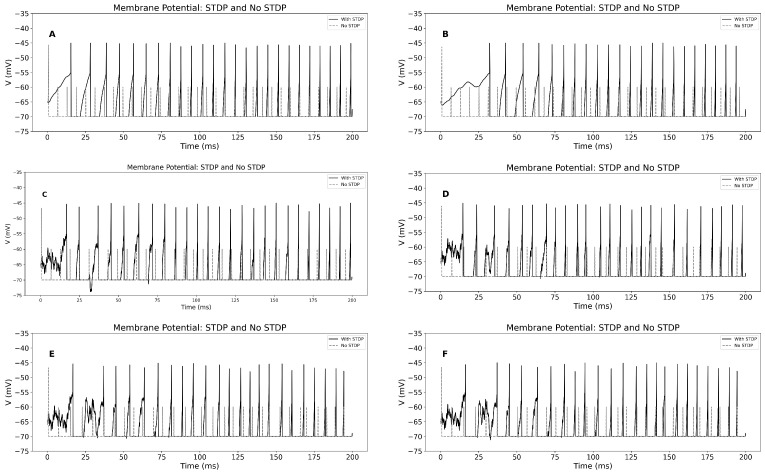
[Color online] Comparison of membrane potential of STDP and no STDP cases. (**A**): The time-dependent changes in membrane potential and synaptic activity profile of direct input current and direct refractory period of healthy cell. (**B**): The time-dependent changes in membrane potential and synaptic activity profile of direct input current and direct refractory period of PD cell. (**C**): The time-dependent changes in membrane potential and synaptic activity profile of random input current σ=1 and direct refractory period of PD cell. (**D**): The time-dependent changes in membrane potential and synaptic activity profile of random input current σ=1 and random refractory period $\sigma_{\mathrm{ref}}=1$ of healthy cell. (**E**): The time-dependent changes in membrane potential and synaptic activity profile of random input current σ=1 and random refractory period $\sigma_{\mathrm{ref}}=1$ of PD cell. (**F**): The time-dependent changes in membrane potential and synaptic activity profile of random input current σ=1, random refractory period $\sigma_{\mathrm{ref}}=1$ and DBS input current of PD cell. Here, we consider $t_{\mathrm{ref}}=6$ (ms).

**Figure 4:**
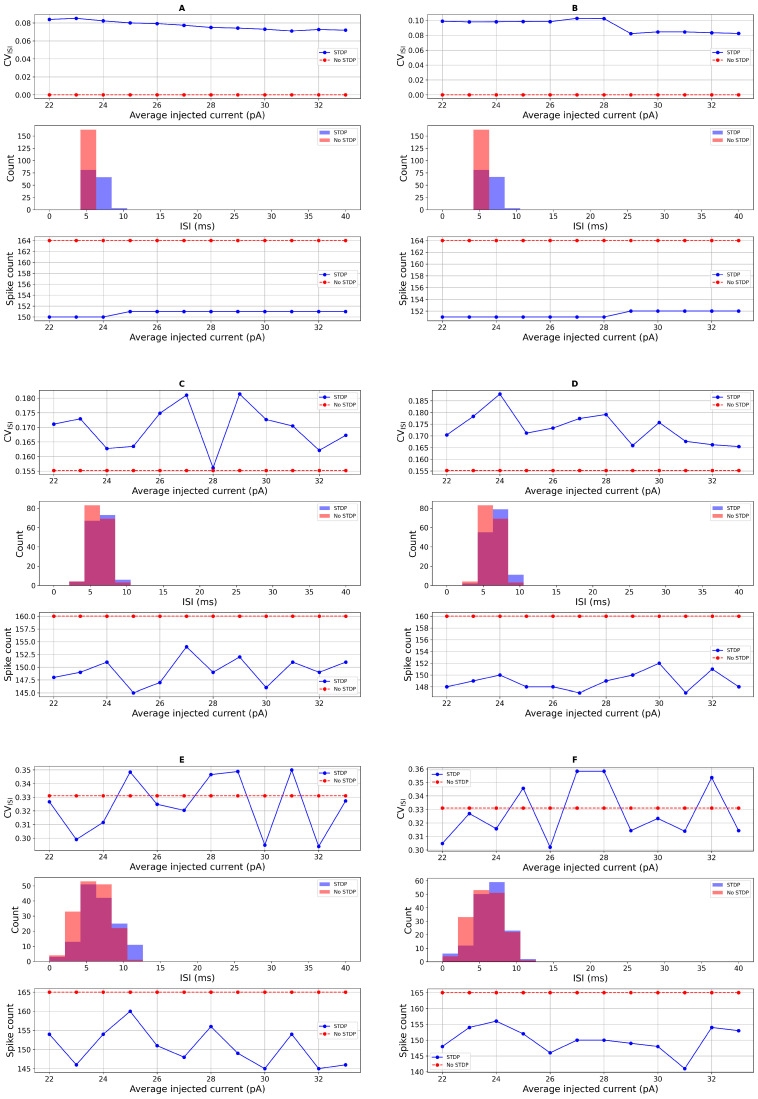
Spike irregularity profiles in the case with the additive noise input current and the ISI distribution. From the top to the bottom, $t_{\mathrm{ref}}=6$ (ms): (**A**): ISI distribution with direct refractory period and direct input current. (**B**): ISI distribution with direct refractory period and random input current σ=1. (**C**): ISI distribution with random refractory period $\sigma_{\mathrm{ref}}=1$ and direct input current. (**D**): ISI distribution with random refractory period $\sigma_{\mathrm{ref}}=1$ and random input current σ=1. (**E**): ISI distribution with random refractory period $\sigma_{\mathrm{ref}}=2$ and random input current σ=2. (**F**): ISI distribution with random refractory period $\sigma_{\mathrm{ref}}=2$ and random input current σ=2 and DBS.

**Figure 5:**
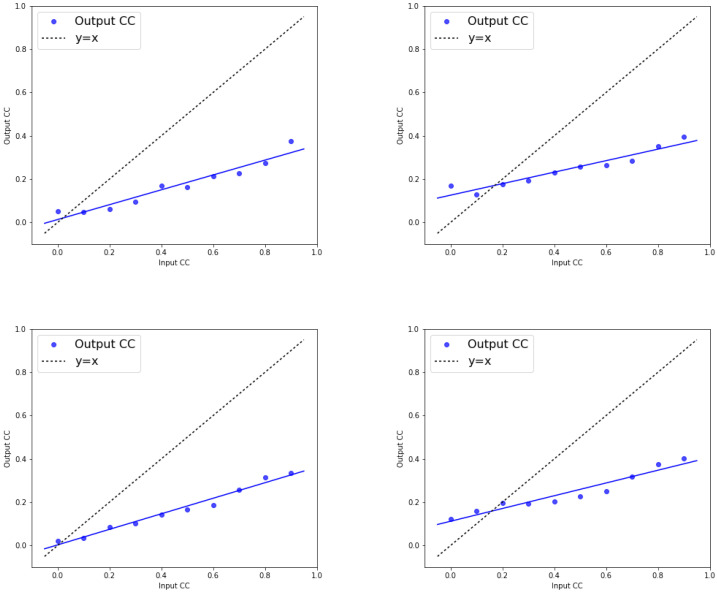
[Color online] Input and output correlations for PD (first row from the top to the bottom) and healthy cell (second row). First column: $\sigma_{\mathrm{ref}}=1$. Right column: $\sigma_{\mathrm{ref}}=4$.

To gain insight into the intrinsic behavior of the model, we first examine its response to randomly generated input currents and synaptic activity under simulated conditions. The main numerical results of our analysis are shown in Figure 2, Figure 3, Figure 4 and Figure 5, where we have plotted the time evolution of the membrane potential calculated based on model (15)–(27), along with the spike count profile, the corresponding spike irregularity profile, and the effects of input correlations on the output correlations for STN healthy and PD cells with STDP. We investigate the effects of additive type of random input currents in the presence of a random refractory period and the input correlations in a modified HH neuron under synaptic conductance dynamics with STDP. We observe that the spiking activity of a neuron in the STN cell membrane potential is influenced by random external currents, random refractory periods, STDP, and input correlations.

In order to switch from healthy conditions to Parkinsonian conditions in the STN model, we consider a decrease in the current $I_{\mathrm{app}}$ applied to the STN. In particular, we have $I_{\mathrm{app}}=33$ (pA) for a healthy STN cell and $I_{\mathrm{app}}=23$ (pA) for a Parkinsonian STN cell (see, e.g., [30]). Therefore, a STN cell in the case of injected current input $I_{\mathrm{app}}=33$ (pA) results in a healthy STN cell, while a STN cell in the case of injected current $I_{\mathrm{app}}=23$ (pA) is considered as a PD-affected STN cell.

In particular, we look at Figure 2A, where we have plotted the time evolution of the STN cell membrane potential with direct input current and direct refractory period of healthy cells. The neuron spikes are different between the neuron spikes in the case of healthy cells and with PD cells presented in Figure 2A,B. There are missing spikes in the time interval of [0,35] (ms) in Figure 2B.

In Figure 2C, in the case of a healthy cell, we added the random input current and random refractory period to the system. We observe that there are fluctuations in the time evolution of the membrane potential (Figure 2C), the total excitatory synaptic conductance (Figure 2C) as well as the trace of the number of postsynaptic spikes over the timescale $\tau_{-}$ (Figure 1B). In the case of PD-affected cells with random input current and random refractory period in Figure 2D–F, we observe that the time evolution of the membrane potential exhibits larger fluctuations and more irregular spiking patterns under these conditions. This is quantified by increased variance in the ISIs and higher membrane potential variability over time. (Figure 2D). There are also fluctuations in the trace of the number of postsynaptic conductance (Figure 2D) and the total excitatory synaptic conductance (Figure 1B).

In Figure 2F, we consider a DBS input to the system. There are still fluctuations in the time evolution of the membrane potential (Figure 2F) and the trace of the number of postsynaptic spikes over the timescale $\tau_{-}$ (Figure 1B).

We also compare the membrane potential in the STDP and no-STDP cases in Figure 3 across various conditions, ranging from direct to noisy inputs. Our observations show that the membrane potential amplitude is higher in the STDP case compared to the no-STDP case.

To further investigate how STDP influences neuronal dynamics, we compared the spiking activity of the neuron model with and without STDP under conditions of additive Gaussian noise in the input current. Our analysis focused on ISI distributions and spike irregularity, as quantified by the coefficient of variation of the ISI ($CV_{\mathrm{ISI}}$). These metrics were evaluated across a range of injected currents, $I_{\mathrm{app}}\in \left[22,34\right]$ (pA), covering both PD and healthy regimes. As shown in Figure 4, the neuron’s output exhibited distinct differences between the STDP and non-STDP conditions. In Figure 4A,B, in the absence of STDP and with minimal noise, the firing behavior of the neurons remains highly regular, resulting in a $CV_{\mathrm{ISI}}$ of zero, indicating no variability in the spike timings. In contrast, spike irregularity was generally higher in the presence of STDP, particularly when noise levels were low or absent. In Figure 4C,D, at $I_{\mathrm{app}},=33$ (pA) (representing a healthy neuron), $CV_{\mathrm{ISI}}$ values increased with STDP compared to the case without STDP, indicating more temporally dispersed spike trains. Similarly, in the PD regime ($I_{\mathrm{app}}=23$ (pA)), neurons with STDP exhibited higher spike irregularity and a broader distribution of ISIs. In Figure 4E, with higher noise, the spike count slightly decreased. However, upon adding DBS input current, the spike count increased. Notably, fluctuations in spike activity due to noisy inputs are visible in Figure 4C–F. The inclusion of a random refractory period, modeled as Gaussian noise in $t_{\mathrm{ref}}$, further amplified the variability in spike timing. This effect was more pronounced in the STDP condition, where the variability in ISI distributions was wider, as reflected in both the histograms and $CV_{\mathrm{ISI}}$ plots. The increased variability suggests that the combination of intrinsic noise and STDP introduces greater temporal complexity in spike generation. Additionally, we observed that the overall spike count was consistently lower in the presence of STDP across all current levels. Despite fluctuations introduced by noise, the firing rate remained suppressed relative to the non-STDP case. This supports the interpretation that STDP introduces adaptive mechanisms that regulate excitability and potentially limit runaway spiking activity.

Together, these results highlight that STDP contributes to increased spike irregularity and reduced spike count, particularly in noisy environments, and that the effects are more pronounced when combined with intrinsic variability such as random refractory periods. These findings are consistent with previous reports, including [18], and support the functional role of STDP in shaping neural response dynamics under both healthy and pathological conditions.

Furthermore, to examine how correlated inputs influence output synchrony in neuronal pairs, we generated pairs of correlated input currents ($I_{1}$, $I_{2}$) and injected them into two separate neurons. For each level of input correlation, we simulated network responses and recorded the resulting output spike times. By repeating this across multiple trials with fixed model parameters and presynaptic spike trains, we computed the output spike–time cross-correlation for each condition. The results presented in Figure 5 are obtained by simulating the network response for varying levels of input correlation (see e.g., Section 2.4). For each input correlation, we compute the output cross-correlation across multiple trials, using a set of predefined model parameters and spike trains for the presynaptic neurons. The plot shows how the input correlation influences the output correlation, reflecting the impact of input correlation on network activity. In Figure 5, the plot of input correlation versus output correlation is referred to as the correlation transfer function of neurons. The results indicate that output correlation is lower than input correlation and output correlation varies linearly with input correlation.

When input correlations do not affect the neurons capacity, output correlation remains independent of both the mean and standard deviation of the refractory period. However, we observe that increasing the standard deviation of the random refractory period in both healthy and PD cells leads to an increase in output correlations, particularly at lower input correlation values (below 0.2). This increase is linear in nature, meaning that as the input correlation increases within this range, the output correlation also increases in a proportional manner. Specifically, this linear trend is most pronounced when input correlations are small, and it reflects how variability in the refractory period amplifies the output correlation as input correlation values approach lower thresholds (see, for example, [70,71]).

#### Sensitivity Analysis of STDP Parameters

**Figure 6:**
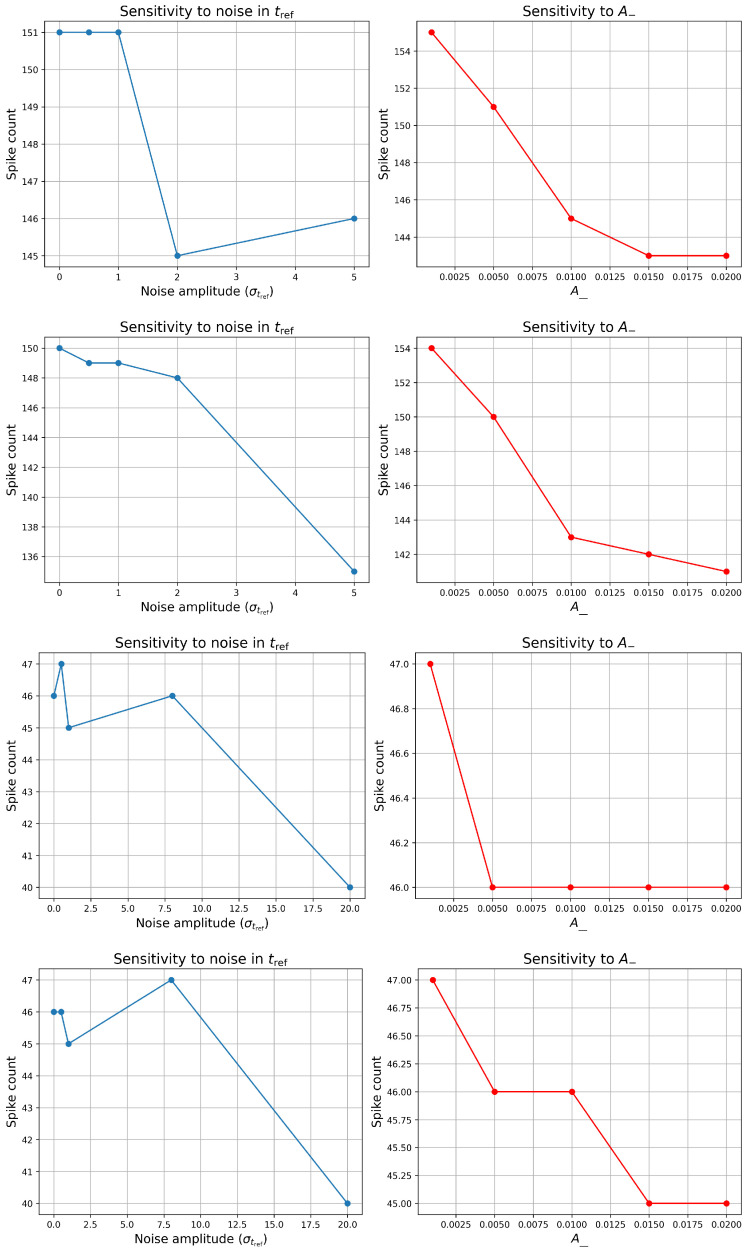
[Color online] Sensitivity analysis of LIF neuron spiking behavior. From the top to the bottom: First row: healthy cell, and second row: PD cell with $t_{\mathrm{ref}}=6$ (ms). Third row: healthy cell, and last row: PD cell with $t_{\mathrm{ref}}=21$ (ms) Left panels: Spike count as a function of Gaussian noise amplitude added to the refractory period ($t_{\mathrm{ref}}$). Right panels: Spike count as a function of the STDP depression parameter ($A_{-}$). The results illustrate how variability in intrinsic timing and synaptic plasticity parameters affect neuronal excitability.

To investigate how variability in intrinsic neuronal properties affects excitability, we conducted a sensitivity analysis by considering Gaussian noise to the refractory periods ($t_{\mathrm{ref}}=6$ (ms) and $t_{\mathrm{ref}}=21$ (ms)) in our model. The value $t_{\mathrm{ref}}=6$ (ms) corresponds to the simulation-based case, while $t_{\mathrm{ref}}=21$ (ms) reflects experimental observations in PD patients; this distinction is discussed in more detail in Section 3.2. Specifically, we evaluated how increasing noise amplitude in $t_{\mathrm{ref}}$ influences the total spike count under different baseline current injection levels representing healthy ($I_{\mathrm{app}}$ = 33 (pA)) and PD ($I_{\mathrm{app}}$ = 23 (pA)) conditions in Figure 6.

In the top left panel of Figure 6, for the healthy neuron, the spike count remained unchanged at 151 spikes for noise amplitudes in the range [0, 1], suggesting robustness to low variability in refractory timing. However, as noise increased from 1 to 2, the spike count slightly dropped to 145, followed by a small increase to 146 across the interval [2, 5]. This nonlinear, U-shaped response may reflect a subtle interplay between the timing jitter and synaptic recovery, where moderate noise disrupts optimal timing, while higher noise introduces compensatory variability that partially restores firing.

In contrast, the PD neuron exhibited a consistent decline in spike count with increasing noise in the bottom left panel of Figure 6. Spiking decreased from 150 at 0 noise to 149 at 1, then to 148 at 2, and markedly down to 135 spikes in the range [2, 5]. This monotonic decline indicates that the PD model is more sensitive to temporal variability, likely due to its reduced excitability and narrower margin for recovery after each spike. The heightened vulnerability to noise in $t_{\mathrm{ref}}$ may reflect impaired temporal precision in pathological conditions. When the refractory period $t_{\mathrm{ref}}$ is increased to 21 (ms), we observe a slight decrease followed by a modest increase in spike count within the noise range [0, 8], after which the spike count declines significantly. This trend is consistent in both healthy and PD model neurons. Interestingly, at a noise level of 8 with $t_{\mathrm{ref}}=21$ (ms), the simulated results closely align with experimental data reported in [72], as discussed further in Section 3.2. However, compared to the case with $t_{\mathrm{ref}}=6$ (ms) and lower noise levels, the overall spiking activity in the $t_{\mathrm{ref}}=21$ (ms) condition is markedly reduced. It is worth noting that the spiking activity observed with $t_{\mathrm{ref}}=21$ (ms) appears more physiologically plausible, whereas the $t_{\mathrm{ref}}=6$ (ms) condition produces an unusually high spiking rate.

These findings suggest that while healthy neurons tolerate moderate intrinsic noise without significant functional loss, PD-like neurons display greater sensitivity, potentially contributing to the disrupted firing patterns observed in PD.

In addition to intrinsic noise, we examined the sensitivity of the neuron’s spiking behavior to changes in the STDP depression parameter, *A*_-_, which governs the magnitude of LTD in synaptic strength following post–before–pre spike pairings. A higher *A*_-_ leads to a stronger weakening of excitatory synapses, reducing postsynaptic excitability over time.

For the healthy neuron ($I_{\mathrm{app}}$ = 33 (pA)), spike count decreased monotonically from 155 to 143 as *A*_-_ increased from 0.001 to 0.02. Similarly, the PD neuron ($I_{\mathrm{app}}$ = 23 (pA)) showed a monotonic decline from 154 to 141 over the same parameter range. A similar phenomenon can be observed in the left panels of the last two rows in Figure 6. This consistent reduction in spike output across both conditions indicates that the neuron’s firing rate is sensitive to the strength of synaptic depression, with stronger LTD leading to progressively reduced excitatory drive.

Interestingly, while both healthy and PD neurons exhibited similar absolute reductions in spike count (∼12–13 spikes), the relative effect may be more functionally significant in the PD case, where baseline excitability is already reduced. This highlights the role of synaptic plasticity in shaping firing dynamics and suggests that maladaptive strengthening of LTD mechanisms could exacerbate firing deficits in pathological states.

Overall, these results underscore that both intrinsic timing noise and synaptic plasticity parameters significantly influence neuronal excitability, with altered sensitivity profiles distinguishing healthy from disease-like conditions.

### 3.2. Comparison with Real Data and Statistical Test

To validate the physiological relevance of the simulated results, we next compare the model’s output with experimental data and perform statistical analysis to assess its consistency with observed neuronal firing patterns. Unlike the results presented in Section 3.1, where we explored the model’s intrinsic dynamics under random inputs, here we adjust and calibrate the model parameters to better fit real electrophysiological data from PD-affected STN neurons in both human and animal studies.

**Figure 7:**
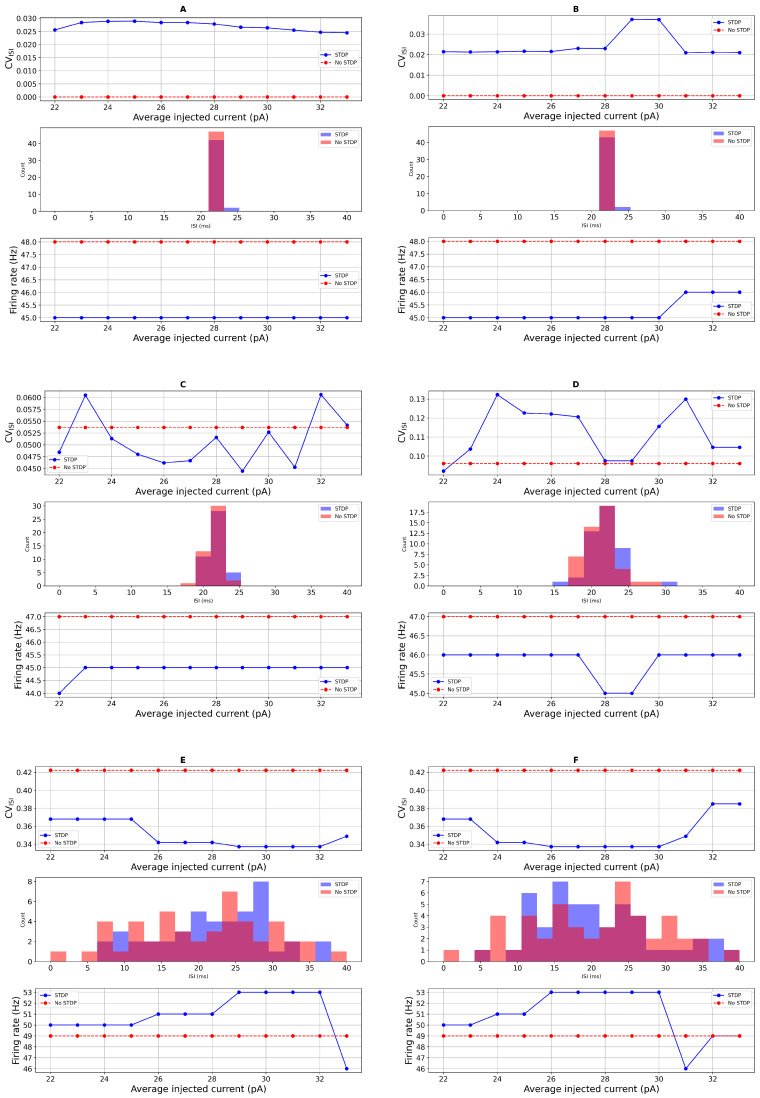
Spike irregularity profiles in the case with the additive noise input current and the ISI distribution. From the top to the bottom, $t_{\mathrm{ref}}=21$ (ms): (**A**): ISI distribution with direct refractory period and direct input current. (**B**): ISI distribution with direct refractory period and random input current σ=1. (**C**): ISI distribution with random refractory period $\sigma_{\mathrm{ref}}=1$ and direct input current. (**D**): ISI distribution with random refractory period $\sigma_{\mathrm{ref}}=2$ and random input current σ=2. (**E**): ISI distribution with random refractory period $\sigma_{\mathrm{ref}}=8$ and random input current σ=8. (**F**): ISI distribution with random refractory period $\sigma_{\mathrm{ref}}=8$ and random input current $\sigma_{\mathrm{app}}=8$ and DBS.

We begin by analyzing spike irregularity profiles generated under additive noise input current and the corresponding ISI distributions, as shown in Figure 4, using a refractory period of $t_{\mathrm{ref}}=21$ (ms). In Figure 7, we increase the noise amplitude and observe a rise in ${\mathrm{CV}}_{\mathrm{ISI}}$ values, particularly when the noise level reaches 8. This trend supports the sensitivity analysis depicted in the bottom row of Figure 6 for the PD condition. Notably, the simulated spike activity aligns well with experimental data from Li et al. (2015) [72], who reported that STN neurons in PD patients exhibit average firing rates of approximately 45±16 (Hz) and ${\mathrm{CV}}_{\mathrm{ISI}}$ around 0.8±0.3 based on single-unit recordings.

**Figure 8:**
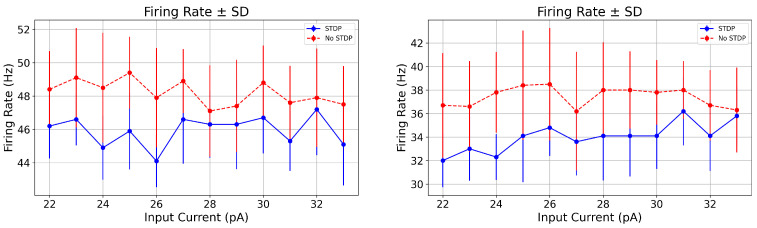
[Color online] Comparison of firing rates with and without STDP across varying input currents. The plot shows the mean firing rate (± standard deviation (SD)) of a STN neuron model as a function of input current amplitude (pA) under two conditions: with STDP (blue) and without STDP (red). Each data point represents the average of 10 simulation trials. Left panel: PD patients. Right panel: Animals.

Our computational model successfully reproduces tonic firing patterns in STN neurons that are consistent with these experimental findings. Under Parkinsonian conditions, the model exhibits firing rates of approximately 46.6±1.56 (Hz) and ${\mathrm{CV}}_{\mathrm{ISI}}$ values ranging from 0.37 to 0.45, as shown in Figure 7 and Figure 8 and summarized in Table 2. These results closely align with the published single-unit electrophysiological recordings, while the experimentally observed firing rate range is broader, the model’s output falls comfortably within those bounds, indicating that it captures a physiologically relevant firing regime for a subset of STN neurons in PD. Thus, both the spike pattern visualizations and the statistical measures support the model’s biological validity.

In contrast, the same study also reported a subpopulation of STN neurons exhibiting highly irregular, burst-like firing with ${\mathrm{CV}}_{\mathrm{ISI}}$ values approaching 0.8 [72]. The relatively lower CVs observed in our simulations suggest that the model primarily represents tonic-firing neurons rather than burst-prone populations. This modeling focus is deliberate, as our aim is to investigate spike timing and synaptic dynamics in stable firing regimes. The regular spiking observed in our simulations is therefore particularly useful for understanding how synaptic inputs and plasticity mechanisms influence temporal precision and firing stability within PD-affected STN networks.

To further examine the impact of synaptic plasticity, we conducted simulations over 10 trials with and without STDP, under both PD (23.0 (pA)) and healthy (33.0 (pA)) input conditions. Here, we consider the case where $t_{\mathrm{ref}}=21$ (ms), $\sigma_{\mathrm{app}}=8$ and $\sigma_{\mathrm{ref}}=8$. Table 2 presents the statistical comparison of firing rate, ${\mathrm{CV}}_{\mathrm{ISI}}$, and spike count across these conditions. Statistical significance was assessed using a two-sample *t*-test [73], with an alpha threshold of α=0.05. In the PD condition, STDP slightly reduced the firing rate compared to the no STDP case (46.6 (Hz) vs. 49.1 (Hz)), with a *p*-value of 0.056, indicating a near-significant trend. More importantly, STDP significantly reduced the ${\mathrm{CV}}_{\mathrm{ISI}}$ (0.348 vs. 0.379, *p* = 0.017), suggesting improved spike timing regularity. These findings, consistent with the spike train patterns and ISI variability shown in the figures, further support the conclusion that our model replicates experimentally observed activity and captures key features of STN firing under PD conditions [72]. In the following, we compare our model outputs with experimental data from PD animal studies.

**Figure 9:**
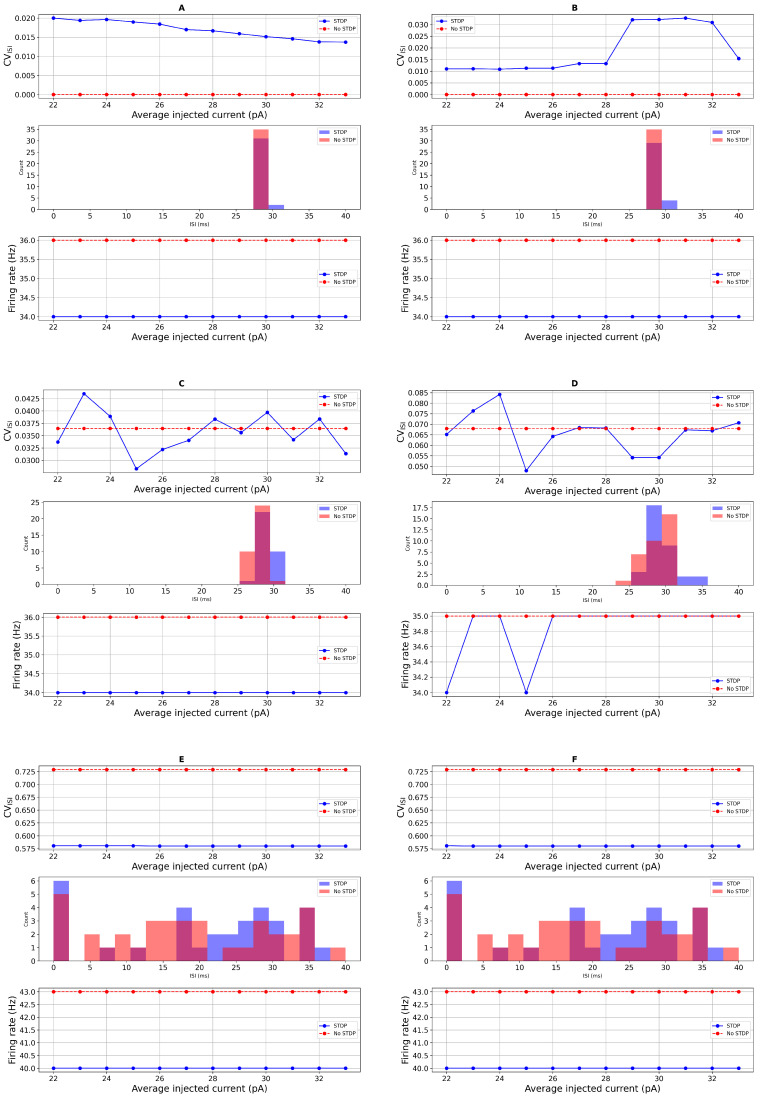
Spike irregularity profiles in the case with additive noise input current and ISI distribution. From the top to the bottom, $t_{\mathrm{ref}}=21$ (ms): (**A**): ISI distribution with direct refractory period and direct input current. (**B**): ISI distribution with direct refractory period and random input current σ=1. (**C**): ISI distribution with random refractory period $\sigma_{\mathrm{ref}}=1$ and direct input current. (**D**): ISI distribution with random refractory period $\sigma_{\mathrm{ref}}=2$ and random input current σ=2. (**E**): ISI distribution with random refractory period $\sigma_{\mathrm{ref}}=18$ and random input current $\sigma_{\mathrm{app}}=18$. (**F**): ISI distribution with random refractory period $\sigma_{\mathrm{ref}}=18$ and random input current σ=18 and DBS.

The firing rates observed in our animal model are consistent with values reported in the literature for PD non-human primates. Specifically, spontaneous STN firing rates around 34.4±16.9 (Hz) have been reported in single-unit recordings from awake, behaving monkeys under certain treatment conditions [74]. In our study, we fix $t_{\mathrm{ref}}=28$ (ms) and the noise levels $\sigma_{\mathrm{app}}=18$ and $\sigma_{\mathrm{ref}}=18$. We observe that the mean firing rates under both STDP and no STDP conditions ranged from approximately 33.0 to 36.6 (Hz) across different input levels (see, e.g., Figure 9 and Table 3), aligning well with the monkeys’ data despite interspecies differences and methodological variability.

While direct ${\mathrm{CV}}_{\mathrm{ISI}}$ values for STN single-unit recordings in non-human primates are scarce, relevant animal studies provide a useful context. In urethane-anesthetized rats, STN neurons exhibit a ${\mathrm{CV}}_{\mathrm{ISI}}$ of approximately 0.65 (with mean firing rates ∼ 5.9 (Hz)) under some control conditions [75]. In macaque vestibular and motor cortex neurons, observed mean ${\mathrm{CV}}_{\mathrm{ISI}}$ measures range approximately 0.344±0.116 during rest or preparatory periods, with dynamic changes tied to behavioral state [76]. Our animal model data, ${\mathrm{CV}}_{\mathrm{ISI}}$ around 0.55–0.60 under no STDP conditions, fall within these published ranges, supporting the biological plausibility of our observations. Moreover, consistent with primate motor cortex findings, the introduction of STDP at lower input levels significantly reduced ISI variability, hinting at a conserved mechanism whereby synaptic plasticity enhances spike train regularity under specific network states.

To further evaluate the impact of STDP on firing dynamics in the animal model under Parkinsonian conditions, we analyzed the simulation outcomes summarized in Table 3. Similar to the human case, we conducted simulations over 10 trials with and without STDP, under both Parkinsonian (23.0pA) and healthy (33.0pA) input conditions. For these simulations, we set the refractory period to $t_{\mathrm{ref}}=28\;\left(\mathrm{ms}\right)$, and used noise levels of $\sigma_{\mathrm{app}}=18$ and $\sigma_{\mathrm{ref}}=18$. At an input current of 23.0 pA, representing a PD excitatory drive, the firing rate decreased from 36.6±3.85 (Hz) (no STDP) to 33.0±2.72 (Hz) (with STDP), with a near-significant *p*-value of 0.059. More notably, the coefficient of variation of inter-spike intervals (${\mathrm{CV}}_{\mathrm{ISI}}$) was significantly reduced from 0.604±0.067 to 0.547±0.060 (*p* = 0.006), indicating that STDP enhances temporal spike regularity in the simulated PD condition. These results were evaluated using a two-sample *t*-test, with statistical significance defined by a threshold of α=0.05. The *t*-test assesses whether the means of two independent groups are significantly different, making it appropriate for comparing the STDP and no STDP conditions in this context.

The changes in spike count closely mirrored the reduction in firing rate, reinforcing the idea that STDP reduces overall excitability while promoting greater spike timing precision. Together, these results suggest that STDP may serve a stabilizing function in modulating subthalamic neuronal activity under pathological conditions, even in non-human primate models. Furthermore, these results align with firing patterns reported in single-unit recordings from awake, behaving monkeys under specific treatment conditions [74].

These results support the conclusion that STDP plays a significant role in shaping both spike rate and temporal dynamics in the STN. In particular, at fixed input noise levels, STDP consistently reduces overall spike output and, under PD conditions, increases temporal variability, potentially reflecting a regulatory mechanism that promotes desynchronization and enhances adaptability in pathological networks.

## 4. Conclusions

In this study, we presented a modified HH model to investigate the interplay between synaptic conductance, random input fluctuations, and STDP in the STN neurons. These elements are known to play a key role in the dynamics of neuronal activity, particularly in the context of neurodegenerative disorders such as PD. By integrating a Langevin-based stochastic framework into our model, we systematically explored how external randomness, through noisy input currents and stochastic refractory periods, affects membrane potential dynamics and neuronal firing patterns. Through a series of simulations, we assessed how the neuron responds to variations in intrinsic noise, STDP parameters, and input correlation structure. Our sensitivity analysis revealed that the spiking behavior is highly dependent on synaptic depression strength and the variability of the refractory period. Specifically, increasing synaptic depression led to a monotonic decrease in spike count, while introducing noise in the refractory period selectively reduced spike count. Additionally, the correlation between the activity of different neurons increases when random inputs and STDP are combined, which further impacts the oscillatory dynamics of the STN.

We also showed that STDP plays a crucial role in modulating both spike rate and temporal irregularity. Compared to the non-STDP condition, STDP consistently reduced spike count and increased ISI variability, suggesting a regulatory mechanism that enhances neuronal adaptability and suppresses excessive synchrony. Furthermore, the correlation transfer analysis confirmed that neurons tend to reduce the strength of incoming correlations, but that this filtering effect can be modulated by intrinsic noise levels, especially through variability in the refractory period.

Importantly, at fixed input noise levels, our model’s firing rates and spike timing variability closely align with experimental recordings from PD patients and animals, with statistical analyses confirming the significant impact of STDP on neural firing regularity. These results strengthen the biological relevance of our approach and highlight synaptic plasticity as a key modulator of pathological neuronal dynamics.

From a biological standpoint, the features included in our model, such as noise in the spike recovery process and temporally asymmetric synaptic plasticity, are well supported by experimental observations. The variability of the refractory period has been attributed to underlying stochastic channel dynamics and heterogeneous cellular properties, while STDP has been extensively documented in multiple brain regions, including the cortex and hippocampus. These biological analogs lend plausibility to our model and its predictions.

Although this study is computational in nature, it opens several avenues for further future experimental validation. For example, in vitro experiments using dynamic clamp techniques could be used to inject correlated input currents into pairs of neurons while varying their refractory dynamics to test the model’s predictions on correlation transfer. Similarly, pharmacological or genetic manipulation of synaptic plasticity pathways could help explore the role of STDP in regulating firing rate and spike–time variability. Investigating these predictions in experimental settings could provide deeper insights into how noise and plasticity contribute to neural computation and dysfunction, especially in disorders such as PD.

In summary, our findings underscore the importance of intrinsic variability and synaptic learning rules in shaping neuronal activity. By integrating biologically grounded mechanisms and systematically exploring their effects, the model offers both explanatory insights and testable hypotheses for future experimental neuroscience research.

In future work, we plan to extend the sensitivity analysis to include a broader range of parameters and explore the effects of network-level interactions, as well as pursue experimental validation to further substantiate the model’s predictions.

While this study focused on single-cell simulations, this modeling strategy is well-suited for isolating the specific contributions of synaptic noise, STDP, and intrinsic neuronal properties to firing dynamics in STN neurons. By avoiding network-level complexities, we were able to systematically assess how individual cellular mechanisms shape spike regularity and responsiveness under varying input conditions. However, it is important to note that network topology, including recurrent connections and modulatory feedback from other basal ganglia structures, may influence these dynamics by introducing synchrony, oscillatory activity, and population-level interactions. Future work will extend this framework to incorporate network architectures to evaluate how circuit-level properties and synaptic plasticity jointly shape emergent activity in both healthy and Parkinsonian states.

## Figures and Tables

**Table 1:** Steady-state functions for channel gating variables and time constants for the different ion channels (see, e.g., [30]).

Current	Gating Variables	Gating Variables	Parameters
$I_{L}=g_{L}\left(v-E_{L}\right)$			$g_{L}=2.25$ (nS)
			$E_{L}=-60$ (mV)
$I_{\mathrm{Na}}=g_{\mathrm{Na}}m_{\infty }^{3}\left(V\right)h\left(V\right)\left(V-E_{\mathrm{Na}}\right)$	$m_{\infty }\left(V\right)=1/\left(1+\exp\left(-\frac{V+30}{15}\right)\right)$	$h_{\infty }\left(V\right)=1/(1+\exp\left(-\frac{V+39}{3.1}\right)$	$g_{\mathrm{Na}}=37$
		$\tau_{h}\left(V\right)=1+500/(1+\exp\left(-\frac{V+57}{-3}\right)$	$E_{\mathrm{Na}}=55$ (mV)
$I_{K}=g_{K}n^{4}\left(V\right)\left(V-E_{K}\right)$	$n_{\infty }\left(V\right)=1/\left(1+\exp\left(-\frac{V+32}{8}\right)\right)$		$g_{K}=45$ (nS)
	$\tau_{n}\left(V\right)=1+100/(1+\exp\left(-\frac{V+80}{-26}\right)$		$E_{K}=-80$ (mV)
$I_{T}=g_{T}a_{\infty }^{3}\left(V\right)b_{\infty }^{2}\left(r\right)r\left(V\right)\left(V-E_{T}\right)$	$a_{\infty }\left(V\right)=1/\left(1+\exp\left(-\frac{V+63}{7.8}\right)\right)$	$r_{\infty }\left(V\right)=1/(1+\exp\left(\frac{V+67}{2}\right)$	$g_{T}=0.5$ (nS)
	$b_{\infty }\left(V\right)=1/\left(1+\exp\left(-\frac{V-0.4}{0.1}\right)\right)$	$\tau_{r}\left(V\right)=7.1+17.5/(1+\exp\left(-\frac{V+68}{-2.2}\right)$	$E_{T}=0$ (mV)
	−1/(1+exp(4))		
$I_{\mathrm{Ca}}=g_{\mathrm{Ca}}c^{2}\left(V\right)\left(V-E_{\mathrm{Ca}}\right)$	$c_{\infty }\left(V\right)=1/\left(1+\exp\left(-\frac{V+20}{8}\right)\right)$		$g_{\mathrm{Ca}}=2$ (nS)
	$\tau_{c}\left(V\right)=1+10/(1+\exp\left(\frac{V+80}{26}\right)$		$E_{\mathrm{Ca}}=140$ (mV)
$I_{\mathrm{ahp}}=g_{\mathrm{ahp}}\left(V-E_{\mathrm{ahp}}\right)\left(\frac{\mathrm{Ca}}{\mathrm{Ca}+15}\right)$			$g_{\mathrm{ahp}}=20$ (nS)
			$E_{\mathrm{ahp}}=-80$ (mV)
$I_{\mathrm{dbs}}=5+5\sin\left(2\pi t\right)$ (pA)			

**Table 2:** Statistical summary of firing rate, ${\mathrm{CV}}_{\mathrm{ISI}}$, and spike count under STDP and no STDP conditions for PD patients.

Input (pA)	Condition	Firing Rate (Hz)	${\mathrm{CV}}_{\mathrm{ISI}}$	Spike Count	FR *p*-Value	CV *p*-Value
23.0 (PD)	STDP	46.6 ± 1.56	0.348 ± 0.027	46.6 ± 1.56	0.056 (ns)	0.017
	No STDP	49.1 ± 2.98	0.379 ± 0.029	49.1 ± 2.98		

**Table 3:** Statistical summary of firing rate, ${\mathrm{CV}}_{\mathrm{ISI}}$, and spike count under STDP and no STDP conditions for animals.

Input (pA)	Condition	Firing Rate (Hz)	${\mathrm{CV}}_{\mathrm{ISI}}$	Spike Count	FR *p*-Value	CV *p*-Value
23.0 (PD)	STDP	33.0 ± 2.72	0.547 ± 0.060	33.0 ± 2.72	0.059 (ns)	0.006
	No STDP	36.6 ± 3.85	0.604 ± 0.067	36.6 ± 3.85		

## Data Availability

The data generated in this study are available from the corresponding author upon reasonable request. The data are not publicly available due to ongoing work builds upon this model.
